# Defining and Measuring Indices of Happiness and Unhappiness in Children Diagnosed with Autism Spectrum Disorder

**DOI:** 10.1007/s40617-022-00710-y

**Published:** 2022-04-12

**Authors:** Devon Ramey, Olive Healy, Emma McEnaney

**Affiliations:** 1 School of Social Sciences, Education and Social Work, Queen’s University Belfast, Belfast, UK; 2 School of Psychology, Trinity College Dublin, Dublin, Ireland

**Keywords:** Quality of life, Measuring mood, Indices of happiness, Autism

## Abstract

Improving quality of life (QoL) is the goal of behavior analytic services, but there can be barriers to assessing the QoL of autistic children due to characteristics inherent in the condition. Given that happiness is a fundamental element of QoL, previous research has relied on behavioral indicators of mood (e.g., smiling, crying) to evaluate the overall QoL of disabled individuals. However, the use of these traditional indices may not accurately reflect the emotional well-being of autistic individuals, who are known to engage in idiosyncratic mood indicators. The current study replicated selected procedures from Parsons et al. (2012) to identify and validate the unique mood indicators of young autistic children. The study showed that individualized indices of happiness and unhappiness could be operationally defined and reliably measured among these children. Key findings and limitations of this study are discussed, and the implications of these findings are presented.

**Supplementary Information:**

The online version contains supplementary material available at 10.1007/s40617-022-00710-y.

There is some research to suggest that individuals on the autism spectrum experience a much lower quality of life (QoL) than nonautistic individuals across the lifespan (Ayres et al., 2018; Egilson et al., 2017; Ikeda et al., 2014; Kamio et al., 2012; Kamp-Becker et al., 2010; Kamp-Becker et al., 2011; Khanna et al., 2014; Kuhlthau et al., 2010; Mason et al., 2018; McConachie et al., 2018; van Heijst & Geurts, 2015). The QoL of autistic people has been found to be negatively affected by factors such as age, comorbid psychiatric conditions, and more substantial support needs (Chiang & Wineman, 2014; Kuhlthau et al., 2010; Mason et al., 2018). Thus addressing these needs, and in turn, improving the QoL of individuals on the autism spectrum is often the rationale for providing behavior analytic services. In fact, changing meaningful behaviors that improve the overall QoL and well-being of individuals has been at the heart of applied behavior analysis (ABA) since the beginning (Baer et al., 1968; van Houten et al., 1988).

With QoL being the focus of behavioral interventions, the need for a valid QoL assessment for individuals with disabilities has become more apparent (Verdugo et al., 2005). For many individuals, QoL is measured through either self- or proxy-reports, using Likert-type rating scales and questionnaires (de Vries & Geurts, 2015; Verdugo et al., 2005). However, there are several issues that can arise when using these types of assessments with autistic individuals. First, questionnaires that solely rely on self-report measures require the individual to have more advanced language skills and the ability to understand abstract questions (Felce & Perry, 1995). Individuals on the autism spectrum, but especially younger people in this population, may have difficulty in interpreting certain items due to the social-communication challenges and inflexible thought patterns that are characteristic of the condition (Tavernor et al., 2013). These individuals can struggle with Likert scales, and due to their social interaction difficulties, autistic children are more likely to respond differently to items that measure key domains such as interpersonal relationships and social inclusion (Tavernor et al., 2013). On the other hand, the use of proxy-reports may not provide an accurate picture of the individual’s QoL, as studies have found that proxy-reports tend to underestimate the overall well-being of individuals on the autism spectrum. Guardians of autistic children will often report significantly lower QoL scores than what their children would self-report (Clark et al., 2015; Egilson et al., 2017; Kamp-Becker et al., 2011; Sheldrick et al., 2012; Shipman et al., 2011; Tavernor et al., 2013). Caregiver reports are also considered more subjective and less meaningful than objective measures (Brown, 2017).

The concept of QoL is multidimensional, but it can be argued that individual happiness or personal satisfaction is one of the most important aspects of life quality (Carr, 2007; Felce & Perry, 1995). Thus, it would seem self-evident that there should be a direct measure of happiness as a QoL outcome during behavioral interventions. The need for an objective measure of mood is even more imperative when evaluating the QoL of individuals with limited communication, or those who have difficulties in expressing their emotions in conventional ways (Dillon & Carr, 2007; Parsons et al., 2012). As QoL indicators, practitioners can use overt measures of mood to help judge the efficacy and social validity of behavioral interventions that are intended to improve the overall well-being of an individual (Toole et al., 2003). If an intervention results in more indices of unhappiness or distress for the individual, it could be modified or eliminated from their behavior plan altogether (Green & Reid, 1999). Yet, despite the potential utility of indices of happiness and unhappiness, mood is not a common dependent variable found within the behavior analytic literature.

There are myriad published studies based on the science of ABA, but a recent review of the literature only yielded 29 studies that have incorporated an objective mood assessment during interventions designed for autistic individuals (Ramey et al., 2019). This paucity of research may be explained by the nature of the field. Behavior analysts are inclined to measure observable (i.e., overt) behaviors, but from a behavioral perspective, happiness is considered a private event that cannot be directly observed (Green & Reid, 1996). Although behavior analysts agree that improving QoL is the objective of ABA, there is an issue with how to measure the emotional well-being of individuals (Pietro et al., 2014). Verbal self-reports are most often used to indirectly measure the private events of others, but those with significant communication difficulties, such as autistic children, will frequently lack the necessary skills to verbally report their emotional experiences (Parsons et al., 2012). Thus, practitioners must find a way to quantify mood in an objective manner. By measuring the overt indicators of happiness and unhappiness theorized to be associated with an individual’s mood (e.g., smiling, crying), researchers can employ a more objective approach to evaluating this behavior.

A prerequisite to measuring happiness is having a valid way of identifying and operationally defining its related mood indicators (Parsons et al., 2012). Green and Reid (1996) first introduced and validated a method for operationally defining indices of happiness and unhappiness among individuals with profound multiple disabilities (PMD). The authors defined these behaviors as “any facial expression or vocalization typically considered to be an indicator of happiness [or unhappiness] among people without disabilities” (Green & Reid, 1996, p. 69). These indices were then confirmed through a two-step validation process and subsequently manipulated through environmental arrangements. The methodology used by Green and Reid (1996) has influenced several other related studies with individuals with PMD or severe/profound intellectual disability (see Dillon & Carr, 2007; Lancioni et al., 2005, for reviews of this literature). However, many of these studies presented with the same limitation. Like Green and Reid (1996), most of the studies measured the same traditional indices seen in those without disabilities (e.g., smiling, crying) across all included participants. This goes against best practice guidelines, which recommend that operational definitions of target behaviors are precisely written to ensure accurate, valid, and reliable measurement (Kahng et al., 2021). As noted by Dillon and Carr (2007), individuals with disabilities may display idiosyncratic mood indicators, which would necessitate individualized behavioral definitions to ensure accurate data collection.

These individualized definitions of happiness and unhappiness are also lacking within the autism literature. For example, of the 29 studies identified by Ramey et al. (2019), only two studies included individualized operational definitions of mood for their participants (i.e., Lattimore et al., 2009; Parsons et al., 2012). Although traditional indices of happiness and unhappiness can be used with some autistic adults, they are not representative of all individuals within this population (Lattimore et al., 2007). Autistic people are known to engage in idiosyncratic sensory and motor behaviors that differ from the general population (Donnellan et al., 2013). It should be noted that their emotional expressivity has been rated as more intense and less natural than their nonautistic peers (Faso et al., 2015). Research has also found that some autistic individuals will engage in noncontextual laughter as a form of vocal stereotypy, which may or may not be associated with their relative degree of happiness (Ahearn et al., 2003; Ahearn et al., 2007; Anderson & Le, 2011; Colón et al., 2012; Gibney et al., 2020; Nikopoulos & Panagiotopoulou, 2015; Wunderlich & Vollmer, 2015). Therefore, it is recommended that operational definitions of mood are individualized for each autistic person so that their idiosyncratic indicators of happiness and unhappiness can be accounted for.

To address this issue, Parsons et al. (2012) first outlined a procedure for identifying and validating individualized indices of happiness and unhappiness among autistic adults who were minimally vocal. The authors found that overt indicators of mood could be operationally defined and reliably measured among this population. Like other overt behaviors, these indices were capable of being systematically manipulated through environmental events. The authors concluded that indices of happiness and unhappiness are important variables to consider when evaluating the outcomes of behavioral services whose overriding goal is to improve the QoL of their consumers (Parsons et al., 2012). There is a need for more literature in this area, but especially with autistic children, as many of the aforementioned studies were conducted with adolescents and adults. Therefore, the aim of the current study was to partially replicate the procedures described by Parsons et al. (2012) with preschool-aged children on the autism spectrum. The purpose of the study was to determine whether individualized indices of happiness and unhappiness could be operationally defined and reliably measured among this younger population.

## Method

### Participant Characteristics

Participants were recruited from a private preschool for children on the autism spectrum located in the Republic of Ireland. To be included in the study, the participants were required to meet the following criteria: (1) have a diagnosis of autism spectrum disorder (ASD) from an independent clinical psychologist; (2) have functional communication skills (i.e., the ability to meet their needs through either vocal speech, the picture exchange communication system [PECS], and/or an augmentative and alternative communication [AAC] device); and (3) be able to select an item between two or more items or pictures.

Nine young boys met the inclusion criteria (see Table 1). The children ranged in age from 3 years 2 months to 6 years 1 month (*M* = 4.7 years). According to school records, six of the participants presented with a secondary diagnosis at the time of the study. Five of the boys communicated with PECS, whereas Daniel and Jack communicated with both PECS and one-word vocal requests. Louis could make requests using short phrases and sentences, whereas Trevor was the only participant that communicated using an AAC device.

**Table 1:** Participant characteristics

Participant	Age (yr:mo)	Sex	Race	SCQ Total Score	Functional Communication	Secondary Diagnosis
Jesse	5:3	M	White	23	PECS	GDD
Daniel	6:1	M	White	21	One-word requests; PECS	ID
Ryan	6:1	M	White	25	PECS	ID
Jacob	3:9	M	White	26	PECS	Right-sided hemiparesis; GDD; LD
Joel	3:4	M	White	19	PECS	None
Louis	4:3	M	White	27	Short phrases and sentences	None
Seth	5:11	M	White	18	PECS	GDD
Jack	3:2	M	White	26	One-word requests; PECS	None
Trevor	4:10	M	White	26	AAC device	ID

yr = year; mo = months; SCQ = Social Communication Questionnaire; PECS = Picture Exchange Communication System; GDD = global developmental delay; ID = intellectual disability; LD = language delay; AAC = augmentative and alternative communication

#### Social Communication Questionnaire

The presence and intensity of the children’s autistic traits were assessed using the Lifetime version of the Social Communication Questionnaire (SCQ; Rutter et al., 2003). The SCQ Lifetime form included 40 items that were completed by each participant’s lead teacher using a dichotomous (i.e., yes/no) format. Item 1 was not scored; rather, it determined the total number of items to be completed based on the language abilities of the child in question. For Items 2, 9, and 19–40, a score of 1 was given for “no” responses, whereas a score of 0 was given for “yes” responses. For all remaining items, a score of 1 was given for the presence of the behavior (i.e., “yes” response), whereas a score of 0 was given for its absence. An SCQ Total Score was obtained by adding items 2–40 for the children who spoke using short phrases or sentences (i.e., Louis), or items 8–40 for the remaining children who did not. According to the manual, a cut-off score of 15 suggested that ASD was present, and the higher the Total Score, the more extensive the support needs were for that child (Rutter et al., 2003). The SCQ Total Scores for the participants are reported in Table 1.

### Setting and Materials

All sessions were conducted in either the participant’s regular classroom or inside the school’s playroom. The classrooms had individual desks and chairs, a large group table, and other items typically found within an early education classroom such as visual supports, toys, and books. The playroom included large play and sensory items such as a slide, trampoline, steamroller, bean bag chairs, and a ball pit. For consistency purposes, other students and teaching assistants were present throughout the study, as changes in routine can inadvertently lead to distress for autistic individuals (Cohen et al., 2010). To avoid interruptions by other students, the teaching assistants were asked to redirect the children away from where the sessions were taking place.

The materials used in each session were unique to each child, as they depended on their individualized happy and unhappy conditions described below. At the end of each session, the researcher used a 30 cm × 18 cm visual aid for the modified self-report measure. This visual had three emojis that represented the emotions *happy*, *okay* (i.e., neutral), and *sad*. The emojis were colored green, yellow, and red, respectively, similar to the emotion thermometer (Attwood, 2006) or the Incredible 5-point scale (Buron, 2021) often used in early education classrooms. All sessions were video recorded with an iPad.

### Dependent Variables and Measurement

The dependent variables for this study were the individualized indices of happiness and unhappiness for each child. These behaviors were identified through the Indices of Happiness and Unhappiness Questionnaire, then subsequently defined through informal observations that were conducted prior to the happy and unhappy conditions. Across both conditions, the researcher observed the video recordings and marked the presence or absence of the happy and unhappy indicators using 10-s partial interval recording. All sessions were 5 min in duration and each session consisted of thirty 10-s intervals. Data were reported as the percentage of intervals with indices of happiness and indices of unhappiness, respectively.

#### Indices of Happiness and Unhappiness Questionnaire

For each participant, three adults who were familiar with the child (i.e., parent, lead teacher, teaching assistant) completed the Indices of Happiness and Unhappiness Questionnaire (Appendix A). This four-item, open-ended questionnaire was developed for this study based on the survey questions utilized by Parsons et al. (2012). The first two questions asked the respondents what specific behaviors their child would engage in when they were deemed to be happy and when they were deemed to be unhappy. The latter two questions asked what type of setting or situation the child was most likely to feel happy and unhappy, respectively.

The responses to the first two questions were compared. Any indices of happiness or indices of unhappiness that were agreed upon by at least two adults were selected for observation. The researcher then conducted 1 or 2 days of informal observation with each participant to confirm the presence of these happy and unhappy indicators. During these observations, the researcher observed the children during their regularly scheduled school activities and noted the topography of their idiosyncratic mood indicators as they naturally occurred. Based on these observations, the indices of happiness and unhappiness were operationally defined for each participant.

For Louis, only two indices of happiness were agreed upon by the adults familiar with him (i.e., giggling and positive talk). However, during his informal observation, the researcher noted that Louis rarely engaged in positive talk, but quite frequently engaged in smiling during preferred activities. As a result, a follow-up questionnaire was given to his respondents to complete. The adults were asked if Louis would smile in situations he was deemed to be happy, and they were expected to respond with “yes,” “no,” or “sometimes.” As all three adults responded with “yes” or “sometimes,” smiling was included as a third indicator of happiness for this participant. The individualized indices of happiness and unhappiness for each participant are operationally defined in Table 2.

**Table 2:** Indices of happiness and unhappiness for each participant

Participant	Indices of Happiness	Indices of Unhappiness
Jesse	*Jumping*: two or more instances of bouncing or jumping in place while in a seated, standing, or kneeling position *Laughing*: giggling and/or chuckling to make an audible noise while smiling *Smiling*: upward curvature of the mouth with or without showing teeth	*Flopping*: lying body down on the floor or table when not asked to do so *Hitting*: using an open or closed hand to strike another person *Screaming*: a high-pitched vocalization above normal conversational level *Crying*: whining and/or wailing accompanied by facial contractions with or without tears
Daniel	*Clapping*: rapidly bringing hands together to generate an audible noise while not engaging in any indices of unhappiness *Laughing*: giggling and/or chuckling to make an audible noise while smiling *Vocalizations*: any audible noise that is not considered a functional word or sentence related to the situation	*Crying*: whining and/or wailing accompanied by facial contractions with or without tears *Hitting*: using an open or closed hand to strike another person; repeatedly slapping the table at a mild to moderate intensity *Flopping*: lying body down on the floor or table when not asked to do so
Ryan	*Laughing*: giggling and/or chuckling to make an audible noise while smiling *Smiling*: upward curvature of the mouth with or without showing teeth *Hugging*: bringing his body close to an adult to receive a hug; wrapping his arms around an adult’s body *Vocalizations*: vowel-like sounds (e.g., “eee,” “ooo,” “ehh”) unrelated to the situation; does not include whistling	*Self-injury*: slapping head with an open or closed hand *Crying*: whining and/or wailing accompanied by facial contractions with or without tears *Bouncing*: two or more instances of bouncing up and down at a high intensity while seated in his chair
Jacob	*Smiling*: upward curvature of the mouth with or without showing teeth *Giggling*: an auditory light laughter accompanied by smiling	*Crying*: whining and/or wailing accompanied by facial contractions with or without tears *Self-injury*: biting hand or banging head off floor if he is laying on the ground *Swiping*: using one or two hands to push items off the table or away from him (including blocked attempts) *Kicking*: using one or two feet to strike another person *Flopping*: lying body down on the floor or table when not asked to do so
Joel	*Smiling*: upward curvature of the mouth with or without showing teeth *Laughing*: giggling and/or chuckling to make an audible noise while smiling *Hugging*: bringing his body close to an adult or leaning on an adult to receive a hug; wrapping his arms around an adult’s body	*Crying*: whining and/or wailing accompanied by facial contractions with or without tears *Flopping*: lying or sitting down on the floor or table when not asked to do so
Louis	*Giggling*: an auditory light laughter accompanied by smiling *Positive talk*: making statements such as “awesome,” “good job,” “that’s funny” *Smiling*: upward curvature of the mouth with or without showing teeth	*Crying*: whining and/or wailing accompanied by facial contractions with or without tears *Hitting*: using an open or closed hand to strike another person or item *Negative talk*: making statements such as “no,” “what’s wrong,” or asking for a tissue repeatedly (e.g., “tissue,” “wipe”)
Seth	*Smiling*: upward curvature of the mouth with or without showing teeth *Hand flapping*: shaking one or two hands either in an up/down or side-to-side motion repeatedly *Jumping*: two or more instances of bouncing or jumping in place while in a standing position *Running*: moving feet quickly in a jogging and/or skipping manner	*Crying*: vocalizations accompanied by facial contractions with or without tears; rubbing eyes and/or covering his ears
Jack	*Smiling*: upward curvature of the mouth with or without showing teeth *Laughing*: giggling and/or chuckling to make an audible noise while smiling	*Screeching*: making a high-pitched “ahh,” “ehh,” or “yay” sound *Flopping*: lying body down on the floor when not asked to do so
Trevor	*Smiling*: upward curvature of the mouth with or without showing teeth *Laughing*: giggling and/or chuckling to make an audible noise while smiling	*Crying*: whining and/or wailing accompanied by facial contractions with or without tears *Bouncing*: two or more consecutive occurrences of bouncing up and down in his chair while seated *Hand flapping*: shaking one or two hands either in an up/down or side-to-side motion repeatedly

#### Reliability

A second trained observer (i.e., the third author) independently watched the video recordings and collected data for at least 26% of sessions within each condition for each participant, in line with the recommendations made by Wolery et al. (2011). Interobserver agreement (IOA) was calculated for both indices of happiness and indices of unhappiness on an interval-by-interval basis. For each 10-s interval, an agreement was marked if both the first author and the third author coded the occurrence or nonoccurrence of the mood indicator. IOA was calculated for both behaviors by dividing the number of agreements by the total number of agreements plus disagreements and multiplying by 100. The mean IOA for indices of happiness across all participants was 91.4% (range: 86.7%–99.2%), whereas the mean IOA for indices of unhappiness was 98.4% (range: 94.2%–100%). In addition to IOA, Cohen’s kappa was calculated to assess the interrater reliability of the mood measures. The mean kappa value across both mood indicators for all participants was 0.80, indicating substantial agreement (Landis & Koch, 1977).

### Experimental Design

There were two conditions for each participant, which closely mimicked their idiosyncratic happy and unhappy situations identified by the questionnaire. To compare the effects of each condition on the children’s indices of happiness and unhappiness, an alternating treatments design without a baseline was employed. The order of the conditions was randomly alternated across sessions, then counterbalanced to ensure that neither condition was conducted for more than two consecutive sessions. The only exception to this was the first session, in which the happy condition was conducted with all participants. As the researcher was unfamiliar to the children, conducting an initial happy session with the participants was deemed appropriate because the presence of the researcher could have otherwise been associated with nonpreferred or aversive activities. This may have inadvertently led to avoidance behaviors in subsequent sessions, regardless of the condition. Two sessions were conducted with each participant daily and they were separated by at least 1 h to control for carryover effects. For Jesse only, the study comprised of 18 sessions due to his availability. For the remaining eight participants, the study comprised of 15 sessions.

### Procedure

#### Happy and Unhappy Conditions

To validate the individualized indices of happiness and unhappiness identified for each participant, the children were exposed to two different conditions that represented their idiosyncratic happy and unhappy situations. These conditions were identified based on the responses to the latter two questions of the Indices of Happiness and Unhappiness Questionnaire. Any setting or situation that was agreed upon by two or more respondents was selected by the researcher. If there was more than one situation identified for either the happy or unhappy condition, the researcher selected the one that required less staff resources. For instance, if a situation required two or more staff members (e.g., going to the playground), it was not selected because it would have limited the availability of the teaching assistants for other students in the classroom. Furthermore, the first unhappy situation identified for both Daniel and Ryan was eliminated due to ethical concerns. Daniel and Ryan were identical twins, and the respondents all agreed that the most common unhappy situation for each child was when the other brother was crying. The researcher could not ethically evoke crying in one brother in order to conduct the unhappy condition with the other. Therefore, this situation was not selected for either of the children and the second agreed upon situation was selected instead.

Sessions were conducted by the teaching assistant who was most familiar with each child. During both conditions, the children were exposed to their idiosyncratic environmental events for a total of 5 min. If a participant was informally observed to be engaging in any of their mood indicators prior to the start of a session, the session was delayed by at least 5 min until the child was displaying neutral behaviors. Both conditions involved naturally occurring situations that the children would regularly encounter in a typical school day. To minimize any distress during the unhappy condition, the sessions were kept brief, and they were to be terminated based on individualized criteria for each participant. For example, the session termination criterion for Jacob was two or more instances of head banging. Although these criteria were put into place, none of the unhappy sessions had to be terminated for any of the participants. The happy and unhappy conditions selected for each participant are listed in Table 3.

**Table 3:** Happy and unhappy conditions identified for each participant

Participant	Happy condition	Unhappy condition
Jesse	Playing with preferred toys	No access to preferred toys
Daniel	Playing with iPad and adult attention	No access to iPad, preferred toys, or adult attention
Ryan	Eating preferred edibles, playing with sensory toys, and adult attention	No access to preferred food, toys, or adult attention
Jacob	Physical sensory activities (e.g., spin chair, trampoline, bouncy ball)	Arts and crafts or cooking activities that got his hands messy
Joel	Playing with preferred toys and adult attention	Structured work tasks at the table; hearing the sound of a SpongeBob SquarePants toy
Louis	Playing with iPad or balloon and adult attention	Working on difficult or acquisition tasks at the table
Seth	Playing in the playroom and dancing to music	Having to share toys with peers
Jack	Playing with preferred toys	No access to preferred toys
Trevor	Playing with iPad or preferred toys	No access to preferred toys

#### Self-Reported Ratings of Mood

According to Green and Reid (1996), the most important consideration when objectively measuring the mood of individuals who cannot self-report their happiness in conventional ways is to ensure that “what is being observed is intended to be observed” (p. 76). In Parsons et al. (2012), the authors implemented a secondary validation measure in the form of a choice comparison to confirm the indices of happiness and unhappiness identified for each participant. During this process, the participants were asked to choose which activity they wanted to engage in (either their happy or unhappy situation), which was immediately followed by direct access to that activity. All participants consistently selected their happy situation.

As the effects of choice were to be examined in a subsequent study with the same participants (i.e., Ramey & Healy, in preparation), the researcher opted for a different secondary validation measure in the current study. To further validate the indices of happiness and unhappiness identified for each participant, the children were asked to rate their mood following each session using a modified self-report measure. Although autistic children can lack the necessary communication skills to express their mood in conventional ways, self-report rating scales can be modified to meet their needs (MacNeil et al., 2009). Therefore, a visual aid was used to support the children during their mood ratings. Although this was a novel visual for the participants, it was similar to other mood visuals and “I feel” charts that were frequently used within the classrooms.

Following each session, the researcher asked, “How do you feel?” while simultaneously presenting the mood scale with the *happy*, *okay*, and *sad* emojis. The children could respond vocally or by pointing to one of the emojis. If the participant did not respond within 5 s, the researcher recorded *no response* on the datasheet. All other responses were marked accordingly. If the indices of happiness and unhappiness were correctly identified for each participant, it was hypothesized that the children’s self-report ratings would corroborate with the mood indicators more frequently observed during each session.

### Data Analysis

The data were analyzed for variability, level, and trend within each condition. In addition to the visual inspection of the graphed data, mean values and ranges were reported for each dependent variable. Furthermore, the percentage of nonoverlapping data (PND) was calculated to help quantify the differences between the two conditions. PND was determined by comparing the two conditions on a point-by-point basis. For example, the first data point of the happy condition was compared to the first data point of the unhappy condition, the second with second, third with third, and so forth (Wolery et al., 2014). If one condition had more sessions due to randomization, some data were left unused for the comparison. In other words, a data point was only included in the quantification if it had a corresponding data point for comparison. PND was calculated by dividing the number of superior data points by the total number of comparisons and multiplying by 100. A PND score of 100% showed clear superiority of one condition over the other (Richards, 2019).

## Results

For all but one participant, the individualized indices of happiness occurred more frequently during the happy condition relative to the unhappy condition. Likewise, for all but one participant, the individualized indices of unhappiness occurred more frequently during the unhappy condition relative to the happy condition.

### Indices of Happiness

**Fig. 1:**
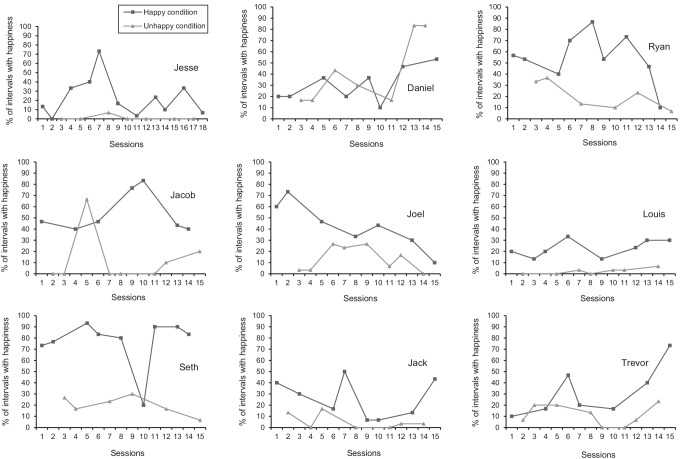
Percentage of intervals with indices of happiness for each participant. *Note.* Dark gray squares represent the happy condition, whereas light gray triangles represent the unhappy condition

The indices of happiness for each participant are displayed in Fig. 1. Jesse demonstrated more indices of happiness during the happy condition (*M* = 23%, range: 0%–73.3%) relative to the unhappy condition (*M* = 1%, range: 0%–6.7%). Although his indices of happiness were highly variable during the happy condition, they were only present at a low level during one session of the unhappy condition. The happy condition was superior to the unhappy condition in six out of seven comparisons, yielding a PND score of 85.7%.

Daniel showed more indices of happiness during the unhappy condition (*M* = 41.4%, range: 16.7%–83.3%) relative to the happy condition (*M* = 30.4%, range: 10%–53.3%). However, there was a highly variable, increasing trend present in both conditions. Although his indices of happiness occurred at a similar level during both conditions, there was a notable increase in these behaviors during the last two sessions of the unhappy condition. The unhappy condition was superior to the happy condition in only four of the seven comparisons, yielding a PND score of 57.1%.

Ryan engaged in more indices of happiness during the happy condition (*M* = 54.4%, range: 10%–86.7%) relative to the unhappy condition (*M* = 20.6%, range: 6.7%–36.7%). Despite the high variability present during the happy condition, his indices of happiness occurred at a moderate-to-high level. Meanwhile, there was a decreasing trend evident during the unhappy condition. There was only one point of overlap between the two conditions, but a PND score of 100% showed clear superiority of the happy condition.

Jacob demonstrated more indices of happiness during the happy condition (*M* = 53.8%, range: 40%–83.3%) relative to the unhappy condition (*M* = 12.1%, range: 0%–66.7%). His indices of happiness were variable in both conditions, but they occurred at a moderate-to-high level during the happy condition. Jacob’s indices of happiness were not present, or they only occurred at a low level, during the unhappy condition. The only exception to this finding was session 5. The happy condition was superior to the unhappy condition in six out of seven comparisons, yielding a PND score of 85.7%.

Joel had more indices of happiness during the happy condition (*M* = 42.4%, range: 10%–73.3%) relative to the unhappy condition (*M* = 13.3%, range: 0%–26.7%). Although there was a decreasing trend during the happy condition, there was only one point of overlap between the two conditions. Joel’s indices of happiness were present at a mostly moderate level during the happy condition, whereas they occurred at a low level during the unhappy condition. There was a PND score of 100%, which showed clear superiority of the happy condition over the unhappy condition.

Louis engaged in more indices of happiness during the happy condition (*M* = 22.9%, range: 13.3%–33.3%) relative to the unhappy condition (*M* = 2.4%, range: 0%–6.7%). His indices of happiness occurred at a low level during both conditions, but they were more frequent during the happy condition in comparison to the unhappy condition. There was no overlap between the two conditions, resulting in a PND score of 100%. This demonstrated clear superiority of the happy condition over the unhappy condition.

Seth demonstrated more indices of happiness during the happy condition (*M* = 76.7%, range: 20%–93.3%) relative to the unhappy condition (*M* = 20%, range: 6.7%–26.7%). With the exception of session 10, his indices of happiness were stable at a high level during the happy condition. During the unhappy condition, his indices of happiness only occurred at a low level. Although there was one point of overlap between the two conditions, the happy condition was clearly superior to the unhappy condition with a PND score of 100%.

Jack had more indices of happiness during the happy condition (*M* = 25.8%, range: 6.7%–50%) relative to the unhappy condition (*M* = 5.2%, range: 0%–16.7%). Although his indices of happiness were highly variable during both conditions, they occurred at a slightly higher level during the happy condition in comparison to the unhappy condition. The happy condition was superior to the unhappy condition, with only one point of overlap between the two. This resulted in a PND score of 85.7%.

Trevor demonstrated more indices of happiness during the happy condition (*M* = 31.9%, range: 10%–73.3%) relative to the unhappy condition (*M* = 11.3%, range: 0%–23.3%). There was an increasing trend present in the happy condition, whereas his indices of happiness remained at a low level during the unhappy condition. The happy condition was superior to the unhappy condition in six out of seven comparisons, yielding a PND score of 85.7%.

### Indices of Unhappiness

**Fig. 2:**
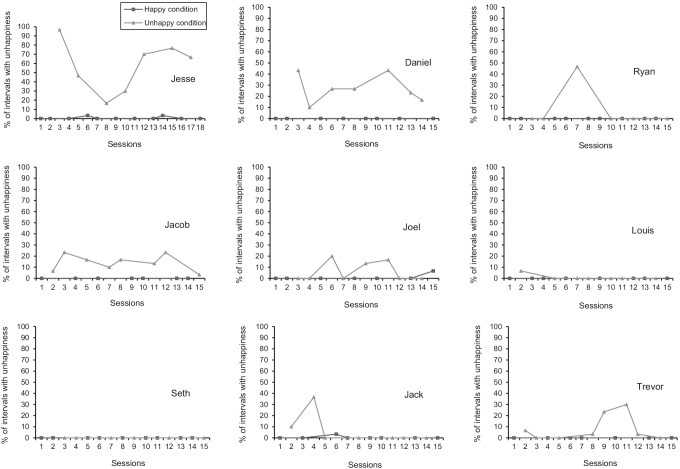
Percentage of intervals with indices of unhappiness for each participant. *Note.* Light gray triangles represent the unhappy condition, whereas the dark gray squares represent the happy condition

The indices of unhappiness for each participant are displayed in Fig. 2. Jesse demonstrated more indices of unhappiness during the unhappy condition (*M* = 57.6%, range: 16.7%–96.7%) relative to the happy condition (*M* = 0.6%, range: 0%–3.3%). His indices of unhappiness were highly variable during the unhappy condition, but they occurred at a moderate-to-high level. Meanwhile, Jesse’s indices of unhappiness only occurred at a low level during two sessions of the happy condition. There was no overlap between the two conditions, resulting in a PND score of 100%. This demonstrated clear superiority of the unhappy condition.

Daniel’s indices of unhappiness were only present during the unhappy condition (*M* = 27.1%, range: 10%–43.3%). During this condition, his indices of unhappiness were variable at a mostly low level. There was clear superiority of the unhappy condition over the unhappy condition, with a PND score of 100%.

Ryan’s indices of unhappiness were rare, as they only occurred during one session of the unhappy condition (*M* = 7.8%, range: 0%–46.7%). During this session, his indices of unhappiness occurred at a moderate level. The unhappy condition was superior to the happy condition in only one of the six comparisons, resulting in a PND score of 16.7%.

Jacob’s indices of unhappiness only occurred during the unhappy condition (*M* = 14.2%, range: 3.3%–23.3%). During this condition, his indices of unhappiness were variable at a low level. The superiority of the unhappy condition was clear, as there was no overlap between the two conditions, which resulted in a PND score of 100%.

Joel had more indices of unhappiness during the unhappy condition (*M* = 6.3%, range: 0%–20%) relative to the happy condition (*M* = 1%, range: 0%–6.7%). His indices of unhappiness were variable at a low level during the unhappy condition, whereas they were only observed during one session of the happy condition. The unhappy condition was superior to the happy condition in only three of the seven comparisons, yielding a PND score of 42.9%.

Louis’s indices of unhappiness were also rare, as they only occurred during one session of the unhappy condition (*M* = 1%, range: 0%–6.7%). During this session, his indices of unhappiness occurred at a low level. The unhappy condition was superior to the happy condition in only one of the seven comparisons, resulting in a PND score of 14.3%.

Seth is the only participant to not demonstrate any indices of unhappiness. The lack of unhappy indicators in either condition resulted in the complete overlap between the two conditions, yielding a PND score of 0%.

Jack demonstrated more indices of unhappiness during the unhappy condition (*M* = 6.7%, range: 0%–36.7%) relative to the happy condition (*M* = 0.4%, range: 0%–3.3%). His indices of unhappiness were rare in both conditions. Nevertheless, Jack’s indices of unhappiness were slightly more frequent during the unhappy condition. During these two sessions, his indices of unhappiness occurred at a low-to-moderate level. The unhappy condition was superior to the happy condition in only two of the seven comparisons, yielding a PND score of 28.6%.

Trevor’s indices of unhappiness only occurred during the unhappy condition (*M* = 8.3%, range: 0%–30%). During this condition, his indices of unhappiness were highly variable at a low level. The unhappy condition was superior to the happy condition in four out of seven comparisons, yielding a PND score of 57.1%.

### Self-Reported Ratings of Mood

Overall, none of the participants consistently responded to the modified self-report measure. When the participants did respond to the mood scale, their ratings seldomly corroborated with the data from the sessions (see Table 4). Four of the children (i.e., Daniel, Ryan, Jacob, Seth) never responded to the self-report measure. Although Jacob responded with *happy* following session 1 of the happy condition, he failed to respond to the mood scale following any of the subsequent sessions. The self-reported ratings of mood for the other five participants were inconsistent. Either the participants did not respond to the question, or their responses rarely validated the observed indices present in the condition.

**Table 4:** Correspondence between self-reported ratings of mood and observed mood indices during happy and unhappy sessions

	Happy condition			Unhappy condition		
Participant	Number of sessions	Responses to mood scale	Sessions with correspondence	Number of sessions	Responses to mood scale	Sessions with correspondence
Jesse	11	8	2	7	6	2
Daniel	8	0	0	7	0	0
Ryan	9	0	0	6	0	0
Jacob	7	1	1	8	0	0
Joel	7	2	2	8	4	0
Louis	8	8	4	7	6	0
Seth	9	0	0	6	0	0
Jack	8	3	0	7	2	0
Trevor	7	0	0	8	4	0

For example, Joel only responded to the mood scale following six of his 15 sessions. Only two of these responses seemed to align with the utilized condition (i.e., *happy* response following the happy condition). A similar outcome was seen in Jack. This participant responded during five of the opportunities, but only two of these responses corresponded with the condition (i.e., *happy* response following the happy condition). However, his indices of happiness during sessions 6 and 9 were observed at a low level. Therefore, it could be argued that these responses did not corroborate the data from these two sessions. Trevor responded to the mood scale following four of the sessions, but these responses did not coincide with the presented condition. During all four opportunities, he responded with *happy* following the unhappy condition.

Jesse and Louis were the only two participants that frequently responded to the modified self-report measure. Both participants responded to the mood scale following 14 of their 18 and 15 sessions, respectively. Louis’s responses corroborated with the observed indices during only four of these opportunities. Although this participant responded with *sad* following session 11 of the unhappy condition, no indices of unhappiness were observed during this session. Finally, Jesse’s mood ratings matched the presented condition during seven of the opportunities. However, for three of these sessions, Jesse responded with *happy* following the happy condition even though his indices of happiness occurred at a much lower level than during sessions 6 and 7. Therefore, only his mood ratings for these two sessions showed correspondence with his observed indices of happiness. It should be noted that both Louis and Jesse often responded with *okay* following their sessions. Whether this was an accurate reflection of their mood during these sessions was unclear.

## Discussion

The purpose of this study was to determine whether selected procedures from Parsons et al. (2012) could be used to identify and validate the individualized indices of happiness and unhappiness of young autistic children. Using the Indices of Happiness and Unhappiness Questionnaire, the researcher identified at least two indices of happiness and one indice of unhappiness unique to each participant. These mood indicators were then validated through a process that involved systematically manipulating the idiosyncratic environmental events identified for each participant. The results for this study corroborated the findings of Parsons et al. (2012), in that eight of the nine participants displayed more indices of happiness during the happy condition relative to the unhappy condition. There was little overlap between the data paths for most of the participants, and the happy condition was considered more superior to the unhappy condition with a mean PND score of 88.9% (range: 57.1%–100%).

Likewise, eight of the nine participants engaged in more indices of unhappiness during the unhappy condition relative to the happy condition. Although the mean PND score was only 51.1% (range: 0%–100%), the superiority of the unhappy condition was evident. For many of the participants, their indices of unhappiness rarely occurred during the unhappy condition, and if they did, they were present at a low level. However, these indices were completely absent during the happy condition for six of the participants. Although the absence of the unhappy indicators during both conditions led to more overlap between the data paths, this finding could be considered more socially valid. The procedures were effective in identifying and validating the unhappy indicators for most participants without needing to evoke high frequencies of these behaviors. Most importantly, none of the unhappy conditions involved contrived aversive situations that the participants would not typically encounter on a daily basis.

It should be noted that Seth’s indices of unhappiness could not be validated, as he did not demonstrate any unhappy indicators during the sessions. One explanation for this finding was that the selected situation for Seth’s unhappy condition (i.e., sharing with peers) was no longer aversive to the participant. It was mentioned by his teacher that sharing with peers was a social skill that the teaching team had previously targeted for improvement, as Seth often engaged in tantrums while playing with peers. To teach this skill, his teaching assistants would praise Seth each time he shared with a peer or cooperated during a game in which he had to take turns. Although this was a socially significant skill to teach, this history of reinforcement likely reduced the aversiveness of the play context. It is possible that the delivery of praise acted as an abolishing operation for Seth’s avoidance behaviors, and in turn, his indices of unhappiness.

The researcher was also unable to complete the secondary validation measure with Seth. In particular, the presentation of the mood scale had inadvertently become an aversive stimulus for this participant, so it was discontinued after session 10. When the visual was presented after the happy sessions, Seth engaged in aggressive and avoidance behaviors that included hitting and running away from the researcher. It was theorized that the visual had become a reflexive conditioned motivating operation (CMO-R; Carbone et al., 2007), because it preceded the termination of his preferred activities and returning to the classroom. This conclusion was supported by the finding that Seth did not demonstrate any avoidance behaviors when he was shown the mood scale following the unhappy sessions.

The findings for Daniel were also unexpected, as he engaged in more indices of happiness during the unhappy condition relative to the happy condition. This could be explained by the nature of Daniel’s unhappy condition. The second unhappy situation agreed upon by his respondents was similar to the alone condition of an experimental functional analysis. This meant that Daniel did not have access to any items or adult attention during the unhappy condition. During these sessions, he was often observed engaging in stimming behaviors, which included hand waving, finger flicking, and sometimes clapping, which was also identified as one of his indices of happiness. It is unclear whether Daniel’s clapping functioned as a self-stimulatory behavior or an indicator of happiness. It is possible that this behavior served both functions, as research has shown that children with and without developmental disabilities will engage in limb stereotypies when they are feeling elated, and when they are understimulated (Willemsen-Swinkels et al., 1998).

Another unanticipated outcome was the decreasing trend in Joel’s indices of happiness during the happy condition. This was likely because an abolishing operation was in effect. Joel had regular access to preferred toys and adult attention outside of the study, and the researcher could not control for how frequently these stimuli were available. With repeated exposure to these stimuli during the happy condition, in addition to his exposure outside of these sessions, Joel may have become satiated. It should be noted that this participant only had two highly preferred items that were previously identified by a preference assessment (i.e., spin chair and toy cars). Although the researcher tried to control for satiation by suggesting that different cars were used across the happy sessions, this may have been ineffective. Frequent access to his preferred stimuli may have inadvertently reduced his indices of happiness, because there is some research to suggest that emotions can be the product of motivating operations (Lewon & Hayes, 2014).

This study differed from Parsons et al. (2012) in a couple of ways. First, the current study did not incorporate a follow-up measure for any of the participants. In Parsons et al. (2012), the happy and unhappy conditions were repeated 12–29 weeks later for one participant and his mood indicators were observed again. These follow-up observations demonstrated intrasubject replication across time, rather than response maintenance after the termination of either condition. As this study included nine demonstrations of intersubject replication, a similar follow-up probe was not deemed necessary. Another difference found within this study was the secondary validation measure used to confirm the individualized indices of happiness and unhappiness identified for each participant. In Parsons et al. (2012), the researchers utilized a choice comparison to further validate the identified mood indicators, whereas this study used a modified self-report as the secondary validation measure. As the effects of choice were to be examined in a subsequent study with the same participants (i.e., Ramey & Healy, in preparation), a choice validation was not included in the present study.

This study has contributed to the autism literature by addressing some of the gaps and limitations found within related studies. First, mood is not often targeted as a primary dependent variable within the literature; it is more commonly measured as a secondary or collateral outcome. Of the 29 studies identified by Ramey et al. (2019), only five studies targeted mood or affect as the primary dependent variable. Another limitation found within these studies is the lack of individualized operational definitions of mood. In addition to Parsons et al. (2012), there was only one other study that measured individualized indices of happiness and unhappiness for each participant (i.e., Lattimore et al., 2009). Across the remaining studies, there was an overutilization of Likert-type mood scales, which are considered less rigorous and more subjective than direct measures such as frequency or interval recording (Ramey et al., 2019). Finally, of the two studies that individualized the operational definitions of mood for each participant, only Parsons et al. (2012) implemented a secondary validation measure to confirm these indices.

The secondary validation measure utilized by the present study was found to be a major limitation, as none of the participants consistently responded to the modified self-report measure. Because of this, the indices of happiness and unhappiness for each participant could not be further validated beyond the idiosyncratic happy and unhappy conditions. Although most of the children were nonvocal, it was hypothesized that the visual aid would help them to communicate their emotions following each session. This assumption was based on the fact that similar mood visuals and “I feel” charts were used within the classrooms, and several of the participants were learning to identify emotions during receptive labeling tasks. Other researchers have reported similar challenges when examining self-reported measures of pain in autistic children with intellectual disability (Fitzpatrick et al., 2020). Furthermore, meta-analyses of self-report measures have shown that emotional self-awareness is diminished in autism (Huggins et al., 2021). This barrier could be explained by alexithymia, which is a common trait in autism characterized by difficulties in identifying and describing one’s own emotions (Griffin et al., 2016; Kinnaird et al., 2019).

Practitioners should be mindful of these difficulties in emotional processing, given that similar self-report measures, such as the Incredible 5-Point Scale (Buron, 2021) and the emotion thermometer (Attwood, 2006), are frequently used in early education classrooms. Before these types of mood scales can be implemented effectively, emotional self-awareness should be taught to young children on the autism spectrum. This requires a targeted behavioral intervention, such as the one described by Fitzpatrick et al. (2020). Future research should explore the efficacy of such self-report measures following a comprehensive social-emotional curriculum that teaches emotional self-awareness to autistic children. By teaching these children how to identify and communicate their feelings, practitioners could have an additional measure of mood to corroborate their objective observations.

As feelings of happiness and unhappiness are a subjective experience, it is recommended that practitioners rely on behavioral indicators of mood within applied settings as this is the most accessible option (Fitzpatrick et al., 2020). Nevertheless, practitioners must acknowledge that indices of happiness and unhappiness can only provide indirect evidence of an individual’s emotions, as mood is a private event that cannot be readily observed. The behavioral indicators in this study were only presumed to correlate with the relative degree of happiness experienced by the children during the sessions. Therefore, any definitive conclusions regarding the children’s moods during the sessions must be made with caution. Future research aiming to improve the happiness of autistic individuals should explore the use of physiological measures (e.g., electrodermal activity) to confirm the link between the observed mood indicators and the private event in question.

In conclusion, the current study demonstrated that individualized indices of happiness and unhappiness can be operationally defined and reliably measured in young autistic children. Like Parsons et al. (2012), this study found that the system for identifying and validating these mood indicators was a relatively simple and time efficient process. However, the measurement system did require more advanced analytical skills, so partial interval recording may not be a suitable method for novice practitioners without appropriate training (Dillon & Carr, 2007; Parsons et al., 2012). One might also question the necessity of such procedures, given that there were traditional indices (e.g., smiling, crying) identified for all participants. Although the researcher could have exclusively relied on these traditional indices, this may have not been a valid measure of mood for the participants on an individual basis.

For example, Joel and Jack frequently engaged in smiling and laughing during their happy sessions, but Jesse and Seth were more likely to demonstrate jumping or running behaviors when they were deemed to be happy. Furthermore, crying was rarely observed because it occurred later within a response class hierarchy for most of the children. In other words, the children were more likely to engage in precursor indices of unhappiness (e.g., flopping, bouncing) before they began to cry. It is also worth noting that the indices of happiness identified for some participants (e.g., hand flapping for Seth), were found to be unhappy indicators for other children (i.e., Trevor). This underscores the importance of individualizing the operational definitions of mood for different children who may not be able to express their emotions in conventional ways.

Most importantly, this study proved that the indices of happiness and unhappiness identified for each child could be systematically manipulated through environmental arrangements. In view of this, these indices could be measured as direct outcomes during intervention. By directly measuring these indices, practitioners have a more objective approach to evaluating the happiness of individuals with disabilities during interventions intended to improve their overall QoL. This is crucial when examining the QoL of individuals who may struggle to communicate their emotions, such as children on the autism spectrum (Parsons et al., 2012). Despite QoL being a goal of support services, it is frequently measured through subjective instruments that often include proxy reports, which can be considered less meaningful than objective measures (Brown, 2017). Furthermore, the social acceptability of interventions intended to help individuals with disabilities is often based upon the opinions of professionals or caregivers, rather than direct measures (Toole et al., 2003). By using this technology, practitioners can evaluate both the efficacy and social validity of interventions intended to improve the overall well-being of autistic children. In fact, this may be one of the few methods that can be used to objectively validate the programming of individuals with disabilities, regardless of their support needs (Ivancic et al., 1997). By using these methods, practitioners can assure that they are practicing in line with the philosophies of person-centered planning and self-determination (Dillon & Carr, 2007).

## Supplementary Information

ESM 1 (DOCX 12 kb)

## Data Availability

The datasets generated during and/or analyzed during the current study are available from the corresponding author on reasonable request.
