# Supplementary material for: Defining and Measuring Indices of Happiness and Unhappiness in Children Diagnosed with Autism Spectrum Disorder
Source: Behav Anal Pract. 2022 Apr 12;16(1):194–209. doi: 10.1007/s40617-022-00710-y (PMC10050627; doi:10.1007/s40617-022-00710-y)
Supplement: Supplementary file 1 — (DOCX 12 kb) [file 40617_2022_710_MOESM1_ESM.docx]

**Appendix A**

Indices of Happiness and Unhappiness Questionnaire

**Instructions:** Please answer the following questions to the best of your knowledge. Provide as much detail regarding your child’s behaviors as you can. If more space is needed, please continue on the back of this page.

1. What specific behaviors does your child engage in when he/she is feeling **happy**?

2. What specific behaviors does your child engage in when he/she is feeling **unhappy**?

3. In what situation(s)/setting(s) is your child most likely to feel **happy**?

4. In what situation(s)/setting(s) is your child most likely to feel **unhappy**?
